# Biofilm as a Key Element in the Bacterial Pathogenesis of Forest Trees: A Review of Mechanisms and Ecological Implications

**DOI:** 10.3390/microorganisms13122649

**Published:** 2025-11-21

**Authors:** Miłosz Tkaczyk

**Affiliations:** Forest Protection Department, Forest Research Institute, ul. Braci Leśnej 3, 05-090 Sękocin Stary, Poland; m.tkaczyk@ibles.waw.pl

**Keywords:** biofilm, bacterial tree pathogens, quorum sensing, extracellular polymeric substances, forest phytopathology

## Abstract

Bacterial diseases of forest trees represent an increasing threat to ecosystem health and the sustainability and resilience of forest management, particularly under changing climate conditions. One of the key yet still insufficiently understood adaptive mechanisms of pathogens is biofilm formation—a structured community of bacterial cells embedded in a matrix of extracellular polymeric substances (EPS), which provides protection against stress factors, biocides, and the host’s defensive responses such as antimicrobial compounds or immune reactions. This paper presents a comprehensive review of current knowledge on the role of biofilms in the bacterial pathogenesis of forest trees, covering their formation mechanisms, molecular regulation, and ecological significance. Four key stages of biofilm development are discussed—adhesion, microcolony formation, EPS production, and dispersion—along with the roles of quorum sensing systems and c-di-GMP-based signaling in regulating these processes. Examples of major tree pathogens are presented, including *Pseudomonas syringae*, *Erwinia amylovora*, *Xylella fastidiosa*, the *Brenneria–Gibbsiella* complex associated with Acute Oak Decline (AOD) and *Lonsdalea populi*. Biofilm formation is shown to play a crucial role in the colonization of xylem, leaf surfaces, and tissues undergoing necrosis, where biofilms may stabilize decomposition zones and support saprophytic–pathogenic transitions. In the applied section, the concept of “biofilm-targeted control” is discussed, encompassing both chemical and biological strategies for disrupting biofilm structure—from quorum-sensing inhibitors and EPS-degrading enzymes to the use of biosurfactants and antagonistic microorganisms. The need for in situ research in forest environments and the adaptation of advanced imaging (CLSM, micro-CT) and metagenomic analyses to tree systems is also emphasized. This review concludes that biofilms are not merely a physiological form of bacterial organization but a complex adaptive system essential for the survival and virulence of pathogens in forest ecosystems. Understanding their functions is fundamental for developing sustainable and ecologically safe phytosanitary strategies for forest protection.

## 1. Introduction

Bacterial diseases of forest trees present a significant threat to the functioning of forest ecosystems and the sustainability and resilience of forest management, particularly under increasing climate-related stress. In recent decades, reports have documented a growing number of new or expanding bacterial pathogens affecting tree species (e.g., *Xylella fastidiosa* Wells et al., *Pseudomonas syringae* van Hall, *Erwinia amylovora* (Burrill) Winslow et al., *Brenneria goodwinii* Brady et al., *Gibbsiella quercinecans* Brady et al. *Lonsdalea populi* Tóth et al.) [1,2,3,4], highlighting the need for a deeper understanding of their infection and survival mechanisms.

A key element of the survival strategy of many pathogenic bacteria is their ability to form biofilms—organised communities of cells embedded in a matrix of extracellular polymeric substances (EPS), which confer numerous adaptive advantages over planktonic forms, which represent free-living, single cells as opposed to surface-attached communities [5]. Despite major advances in understanding biofilm-mediated pathogenesis in agricultural and crop systems, the role of biofilms in the pathology of forest trees remains largely underexplored and underestimated [6,7].

A biofilm is defined as a structurally and functionally organised microbial community attached to a surface (e.g., bark, leaves, vascular tissues) or residing within host structures, surrounded and protected by a shared matrix composed of polysaccharides, proteins, extracellular DNA, and other EPS components [6]. Biofilm formation typically proceeds through four main stages: (i) initial adhesion, (ii) microcolony formation, (iii) EPS matrix elaboration and biofilm stabilisation, and (iv) cell dispersion into the planktonic phase [8,9,10]. The regulation of these stages is primarily controlled by quorum sensing systems, modulation of signalling molecules (e.g., N-acyl homoserine lactones, DSF), as well as host physicochemical factors and hydrodynamic conditions.

In forest trees, biofilm formation may enable pathogens to persistently colonise leaf surfaces, bark, and xylem vessels, increasing resistance to stressors (desiccation, UV radiation, plant defensins), reducing the effectiveness of chemical agents (plant defensins or other antimicrobial compounds), and promoting the maintenance of chronic infections [3,11].

A model example in which biofilm formation plays a central role in plant disease is *X. fastidiosa*. This bacterium colonises the xylem vessels of its host and produces a biofilm that clogs water-conducting tissues, restricting sap flow and leading to wilting and tissue necrosis. Studies have shown that EPS polymers are essential for biofilm formation, virulence, and vector transmission by insects [8], and that modulation of DSF (diffusible signal factor) regulation is crucial for maintaining the balance between the biofilm and dispersal phases [12]. Furthermore, modelling studies have described a mechanical model of biofilm behaviour within xylem vessels based on polymer dynamics [3]. An intriguing discovery was the identification of morphological plasticity in *X. fastidiosa* cells, where filamentous cell forms emerge prior to biofilm maturation, potentially influencing the spatial organisation of the biofilm [13].

Similarly, in pathogenic *Erwinia* species (e.g., *E. amylovora*), biofilm formation within plant conduits (mainly xylem) is recognised as an important virulence mechanism. The *E. amylovora* biofilm participates in the colonisation of vascular tissues and the establishment of persistent infection sites. The review by Piqué et al. [14] identifies polysaccharides (amylovoran, levan) as key EPS components, along with factors related to motility, quorum sensing, and protein regulators. Moreover, Koczan et al. [15] demonstrated that cell surface structures (e.g., pili, adhesins) significantly contribute to the adhesion and biofilm formation ability of *E. amylovora*.

It is important to emphasise that quorum sensing (QS) mechanisms are widely used by phytopathogenic bacteria to regulate the expression of virulence factors, metabolism, and biofilm formation [16]. In *Erwinia*, the presence of both AHL (N-acyl homoserine lactone) signals and AI-2 (autoinducer-2) molecules indicates complex regulation of cellular communities [17]. Similarly, in Dickeya species, integrated QS networks influence multiple biological functions, demonstrating the universality of signalling strategies in plant pathogenesis [18].

Although the plant pathology literature provides several examples of biofilm formation in the context of crop infections (for example, in orchards and vineyards) [19], relatively few studies have addressed forest microenvironments, which provide highly variable physical and chemical conditions affecting biofilm stability. In particular, there is a lack of in situ research, which is crucial for observing biofilm development directly in natural tree tissues [20]. Moreover, a key technical challenge remains: how can biofilms within the xylem be effectively detected? Microscopic methods (such as CLSM, FISH, and microtomography) require substantial adaptation to the specific structural and chemical conditions of woody tissues.

Drawing on knowledge from agronomic systems and model microbiology, and integrating these concepts with forest ecology, appears to be a promising direction. Therefore, the objectives of this paper are to: (i) review the mechanisms of biofilm formation in tree pathogens; (ii) analyse species-specific examples (such as *Xylella*, *Erwinia*, *Pseudomonas*, *Brenneria*/*Gibbsiella*, *Lonsdalea*); (iii) propose a conceptual model integrating the role of biofilm with tree pathogenesis and forest ecological factors; and (iv) discuss the potential of “biofilm-targeted” strategies in forest phytopathology while identifying key research gaps.

Through this approach, the study aims to highlight a largely underestimated yet potentially crucial component of bacterial pathogenesis in forest ecosystems—biofilm formation—and to inspire novel research and applied approaches for developing sustainable forest protection strategies.

## 2. Biofilm Formation Mechanisms and Their Significance for Tree Pathogens

The formation of biofilms by pathogenic bacteria is an adaptive strategy that enables survival and proliferation under unfavourable environmental conditions. In the context of tree pathogens, these mechanisms may play a pivotal role in the colonisation of leaf surfaces, bark, and particularly vascular tissues. The following section discusses the mechanisms of adhesion and EPS matrix formation, as well as the adaptive significance of biofilm architecture, including the regulation of this process and its implications for bacterial pathogenicity in trees.

### 2.1. Adhesion, Microcolony Formation, and EPS Production

Primary adhesion of planktonic cells to surfaces (leaves, bark, vessel walls) usually occurs through physicochemical forces—van der Waals interactions, electrostatic forces, and hydrophobic interactions. Planktonic cells are free-living and motile, whereas sessile cells are surface-attached and form structured biofilms. At this stage, attachment is often reversible. Only after the production of adhesins, surface proteins, fimbriae, or polysaccharides does the transition to irreversible adhesion and microcolony formation (adhesive clusters) occur [21]. In phytopathogenic bacteria, up to 75–90% of the biofilm volume consists of the EPS (extracellular polymeric substances) matrix, while only 10–25% comprises bacterial cells, highlighting the matrix as the primary structural and protective element [22].

In the pathogen *X. fastidiosa*, nanometre-resolution microscopy has shown that during the initial adhesion phase, polar protein domains initiate cell attachment, after which loosely attached EPS connects microcolonies. Over time, the EPS matrix forms filaments that provide mechanical support to the biofilm and enable its maintenance even under plant sap flow conditions [23]. Additionally, EPS polymers in *Xylella* are crucial for virulence and the ability to block xylem vessels [24].

In *P. syringae* and other bacterial plant pathogens, bacteria possess gene sets for various polysaccharides (e.g., alginate, cellulose, Psl, and other analogous glycans). These operons are often environmentally induced—by nutrient limitation, water stress, or surface perturbations—providing adaptive flexibility and redundancy in biofilm formation. The loss of a single locus rarely abolishes biofilm-forming ability due to the functional redundancy of other operons [22].

Although motility is often reduced in mature biofilms, it plays a critical role in initial surface colonisation—sliding, type IV pili, or flagella help cells reach favourable microenvironments and explore space prior to permanent attachment [22]. In many bacteria, increased levels of cyclic-di-GMP suppress motility while simultaneously activating the expression of EPS and adhesin genes. Two-component systems and transcriptional regulators respond to external cues (e.g., stress, calcium, iron) and modulate transitions between planktonic and sessile phenotypes. Such mechanisms have been described in several plant-associated bacteria [25].

### 2.2. Adaptive Significance of Biofilm: Survival, Resistance, and Colonization

The biofilm structure offers a range of adaptive benefits for tree pathogenic bacteria. Primarily, the EPS matrix acts as a buffer against abiotic stresses: under conditions of periodic drought, UV radiation, temperature fluctuations, and osmotic stress, the biofilm creates a microenvironment that maintains moisture and limits direct exposure of cells. In plant–microbe systems, it has been observed that biofilm-forming pathogens can survive stress periods longer than their planktonic pathogens—i.e., bacteria existing in a free-floating state before adhesion [22].

Another important aspect is protection against host defence mechanisms. The biofilm can reduce the penetration of toxic compounds produced by the plant (e.g., defensins, reactive oxygen species, phenolics) as well as degradative enzymes. In forest tree species, antimicrobial defence chemistry is particularly complex and potent, imposing strong selective pressure on microbial colonizers. Many broadleaf and coniferous trees produce high levels of polyphenolic and resinous compounds, including hydrolysable and condensed tannins, stilbenes, flavonoids, terpenoids, and resin acids, which exhibit strong bacteriostatic or bactericidal activity [26,27,28]. For example, oak heartwood and bark are rich in ellagitannins and gallic acid derivatives that inhibit bacterial quorum sensing and cell wall synthesis [29], while conifers exude oleoresins and monoterpenes (e.g., α-pinene, limonene, abietic acid) capable of disrupting membrane integrity and oxidizing microbial proteins [30,31]. These compounds accumulate in xylem, bark, and resin ducts, often creating locally toxic microenvironments that planktonic bacterial cells cannot tolerate. The extracellular polymeric matrix of biofilms, however, can act as a physical and chemical buffer, reducing diffusion of these lipophilic or oxidizing molecules and enabling pathogens to persist within resin- or phenolic-rich tissues. Consequently, the ability to form biofilms may represent an essential adaptation for bacterial pathogens to survive within chemically defended forest tree tissues—a feature that may explain the relative scarcity of planktonic infections and the chronic nature of biofilm-associated diseases in woody hosts [32]. Within the biofilm, cells display altered expression of stress-response and DNA repair genes, enhancing survival against host attack. In the context of vascular phytopathogens, biofilm formation is recognised as a key strategy to avoid elimination by host defences and environmental stressors [33].

In trees, particularly within vascular tissues, biofilm formation has significant physiological consequences: persistent colonisation of the xylem can lead to vessel blockage, reduced sap flow, wilting symptoms, hydraulic stress, and tissue necrosis. In a theoretical model, Walker et al. [3] used a polymer-based approach to describe biofilm behaviour in olive tree vessels infected with *Xylella*, providing insight into growth dynamics and hydraulic blockages in vascular structures. Observational studies of *Xylella* confirm that the bacterium produces biofilm within vessels and on internal surfaces, correlating with disease symptoms [34].

Biofilms also increase resistance to chemical (e.g., biocides, pesticides) and biological (e.g., antagonistic microbes) agents. Nutrient and oxygen gradients within the matrix favour subpopulations of slow-growing cells that are less susceptible to biocides [33]. Additionally, EPS serves as a physical barrier to degrading enzymes or antimicrobial compounds. In *Xylella*, treatment with N-acetylcysteine (NAC) to degrade EPS demonstrated that matrix removal facilitates cell release and biofilm disruption, underscoring the protective role of EPS [35]. Furthermore, biofilm formation enhances colonisation of leaf and bark surfaces. Bacteria within biofilms can establish microhabitats with favourable water conditions, supporting epiphytic survival and increasing the likelihood of penetration through wounds or natural openings. In plant pathology literature, biofilm formation is considered a critical stage bridging survival and infection [22].

The regulation of biofilm formation is dynamic and complex. Quorum sensing (QS) systems play a central role in coordinating EPS production and activating degradative enzymes when required [36,37,38]. In *Ralstonia* pathogens, for example, carbohydrate-binding lectins and EPS interact depending on environmental conditions to regulate adhesion and dispersal [39]. Dispersal can be triggered by internal signals (e.g., nutrient limitation, QS cues) or external stresses (fluid flow, shear forces), enabling bacteria to colonise new sites. Mathematical biofilm models often consider feedback between sessile and planktonic phases [40].

In contrast to annual agricultural crops, forest trees possess distinctive anatomical and ecological traits that significantly affect biofilm formation and persistence. Mature forest trees have a markedly thicker and more heterogeneous bark structure, complex vascular anatomy, and extended lifespans, all of which create diverse microhabitats for microbial colonization [41,42]. The long-term stability of woody tissues and limited renewal of xylem and phloem favour the establishment of chronic bacterial populations and multilayered biofilms [43,44]. Additionally, the forest tree microbiome—comprising epiphytic, endophytic, and rhizospheric communities—forms a highly complex and competitive network that can either suppress or facilitate pathogen biofilm development depending on environmental conditions [45,46]. Compared with agricultural crops, forest trees also experience slower wound healing and reduced chemical control accessibility, leading to more persistent infections once biofilms are established [2]. These features collectively indicate that biofilm formation in forest trees may be governed by unique ecological constraints and may require specifically adapted control strategies that account for tree age, tissue architecture, and microbiome interactions. An emerging example is *L. populi*, whose bark-associated biofilms illustrate how extracellular exudates can couple pathogen survival with insect-mediated dissemination.

In summary, biofilm mechanisms—from adhesion, EPS production, QS regulation, and motility to dispersal—constitute a comprehensive adaptive system that allows tree pathogens to survive, colonise surfaces and internal tissues, evade host defences, and resist control measures. Understanding these mechanisms in tree pathogens provides a foundation for future research on biofilm control in forest ecosystems.

## 3. Biofilms in Specific Tree Pathogens

Biofilm formation has been reported across a broad range of bacterial pathogens associated with forest and plantation trees [20,47]. These microorganisms differ markedly in their preferred ecological niches, infection strategies, and biofilm architectures, ranging from epiphytic surface films to vascular and necrotic tissue biofilms. Table 1 provides a comparative overview of the main tree-associated bacterial pathogens, summarizing their host range, anatomical localization of biofilm formation, key matrix components, and ecological relevance. This framework serves as a reference for the following subsections, which examine individual taxa in more detail, emphasizing the diversity of biofilm functions in the context of tree pathogenesis and forest ecology.

### 3.1. P. syringae—Epiphytic Biofilms on Leaves and Shoots

In forest ecosystems, *P. syringae* displays a broad host range among both deciduous and coniferous trees, representing a significant phytopathogen in temperate climate zones. The most commonly reported host species are *Prunus avium*, *Aesculus hippocastanum*, *Betula pendula*, *Populus* spp., and *Acer pseudoplatanus* [48,49]. Among conifers, infections have been observed on *Pinus sylvestris* and *Larix decidua*, although typically with lower severity [50]. In forest environments, these bacteria frequently colonise the surfaces of leaves, buds, and bark as epiphytes, transitioning to a pathogenic state in response to plant stress or frost damage. The ability to form biofilms on tree organs enhances survival under adverse conditions, such as high humidity and fluctuating temperatures, which can lead to disease recurrence in spring [51].

*P. syringae* is a typical epiphytic pathogen whose life cycle involves prolonged residence on leaf and shoot surfaces before initiating infection [52]. Many strains can form stable, multilayered biofilms on the leaf cuticle, where the EPS matrix interacts with adhesin proteins and type IV pili, strengthening attachment and microcolony formation [53]. Regulatory mechanisms include quorum sensing systems and c-di-GMP-based signalling, which modulate the switch between motile and sessile phenotypes. Molecular and genetic studies indicate the presence of multi-locus polysaccharide operons (e.g., Wss/Psl/cellulose-like synthases), whose induction is environmentally dependent and provides an adaptive advantage for the epiphytic lifestyle [1,54].

The role of *P. syringae* biofilms in forest survival is multifaceted. The EPS matrix creates localised microenvironments with enhanced water retention, reducing desiccation following sun exposure. Additionally, biofilm structures partially protect cells from UV radiation and temperature fluctuations typical of the tree canopy [55]. Field ecological studies and laboratory models have shown that biofilm formation enhances recolonisation after rainfall and improves overwinter survival, increasing the likelihood of disease foci initiation under favourable conditions [56].

### 3.2. Erwinia spp.—Biofilm Formation in Vascular Tissues

Although *E. amylovora* is primarily known as a pathogen of *Rosaceae* in orchards, it is increasingly identified in forest environments and wooded areas, where it infects wild tree and shrub species that serve as natural reservoirs for the bacterium. Potential hosts in forest ecosystems include *Crataegus monogyna*, *Sorbus aucuparia*, *Amelanchier ovalis*, as well as certain naturally occurring species of *Malus* and *Pyrus* [57].

In *E. amylovora*, biofilm formation plays a critical role in colonising vascular tissues. Biofilm structures within the xylem and adjacent mesophyll spaces facilitate the establishment of persistent infection foci, while three main polysaccharides—amylovoran, levan, and cellulose—have been identified as key EPS components, determining the architecture and viscosity of the matrix [58]. Mutants lacking one or more of these components show reduced biofilm-forming ability and diminished virulence in planta. Numerous genetic and physiological studies also indicate the importance of phosphodiesterases and c-di-GMP regulation in controlling EPS biosynthesis and biofilm dynamics [9,58].

**Table 1:** Overview of Major Bacterial Tree Pathogens and the Characteristics of Their Biofilms.

Pathogen	Common Hosts	Biofilm Location	Main Components	Regulation/Signaling	Functional Consequences	References
*P. syringae*	Deciduous and coniferous trees: *Prunus*, *Aesculus*, *Betula*, *Populus*, *Acer*, as well as *Pinus* and *Larix*.	Epiphytic biofilms on leaf cuticles, buds, bark; microcolonies on surfaces.	Cellulose-type polysaccharides/Psl-like/alginate analogs; type IV pili; adhesive proteins.	c-di-GMP, quorum sensing; multi-locus regulation of EPS operons dependent on environmental conditions.	Provision of water retention, UV protection, increased overwintering survival, higher likelihood of infection initiation after rainfall.	[55]
*E. amylovora*	Mainly Rosaceae: *Malus*, *Pyrus*, *Crataegus*, *Sorbus*, *Amelanchier* (also occurring in forested areas).	Biofilm in vascular tissues (xylem/phloem) and adjacent mesophyll; foci within the vessels.	Amylovoran (major), levan, cellulose; polysaccharide capsule.	Regulation of EPS biosynthesis by systems (including c-di-GMP; genetic regulators such as csrD, etc.).	Vessel occlusion, wilting, and necrosis; EPS acts as a barrier against host defenses and protective agents; EPS-deficient mutants lose virulence.	[15]
*X. fastidiosa*	Broad spectrum: olive, grapevine, citrus; numerous forest species (*Quercus*, *Acer*, *Platanus*, *Ulmus*).	Biofilm in xylem vessels (inner surface of the vessels).	Complex EPS (β-1,4-endoglucanase and other polysaccharides); adhesive proteins; filamentous matrix elements.	DSF (diffusible signal factor) systems; EPS-modifying enzymes (e.g., β-1,4-endoglucanase) regulating polymer length and biofilm dynamics.	Vessel occlusion—reduced sap and water flow, wilting/death; DSF regulation affects biofilm/dispersion balance and vector-mediated transmission.	[8]
AOD complex	Oaks—also widely distributed across Europe.	Biofilms in necrotic tissues under the bark and in necrotic cavities; potential foci in vessels.	Probable multi-species EPS consortium (various polysaccharides); indirect evidence from isolations and metagenomics.	Likely interspecies signaling; consortial interactions; detailed genetic characterization of EPS is lacking (knowledge gap).	Potential stabilization of infection foci in wounds/necroses; protection against host defenses; facilitation of tissue degradation by cooperating enzymes.	[59]
*L. populi*	*Populus* spp. (black poplar, hybrid poplars)	Cortical tissues, bark exudates, wound margins	EPS rich in polysaccharides and proteins; creamy extracellular matrix	Quorum sensing (AHL-like); EPS secretion genes	Protective barrier against desiccation; insect attraction; enhanced bark colonisation and spread	[60]
Other, e.g., *Dickeya*, *Ralstonia*, *Xanthomonas*	Various trees and plants; in forest contexts—mainly a reservoir in the understory and woodlands.	Epiphytic or vascular biofilm depending on the species.	Diverse EPS polysaccharides, eDNA, adhesive proteins—mechanistic community of biofilm traits.	QS (AHL, DSF, etc.), c-di-GMP; EPS-degrading enzymes regulate dispersion.	Facilitates survival, concealment from host defense mechanisms, and increased tolerance to biocides.	[22]

Functionally, *Erwinia* biofilms in vascular tissues mechanically impede water transport (vessel blockage), promote local hydraulic stress, and contribute to wilting and necrotic symptoms [61]. Additionally, EPS can act as a barrier against plant protection agents and natural host defences, complicating pathogen elimination. Ecological reviews of *E. amylovora* further highlight the significance of microenvironmental interactions and redundant EPS systems for pathogen survival and dispersal [17,62].

### 3.3. X. fastidiosa—Biofilm in Xylem Vessels; DSF and the Adhesion–Dispersion Cycle

*X. fastidiosa* is one of the most serious potential threats to forest trees in Europe due to its broad host range and its ability to colonise the xylem of numerous deciduous tree species. In addition to well-documented infections in olive, grapevine, and citrus, this bacterium can colonise many forest species, including *Quercus* spp., *Acer pseudoplatanus*, *Platanus orientalis*, *Ulmus minor*, and *Cercis siliquastrum* [2,63]. Studies conducted in the Mediterranean region have revealed asymptomatic infections in multiple trees growing in forests and wooded areas, suggesting that natural reservoirs of the pathogen exist outside agricultural crops [64]. If *X. fastidiosa* were introduced into Central European forests, there is a risk of permanent colonisation of native tree species, particularly under favourable climatic conditions and in the presence of insect vectors from the genera *Philaenus* and *Neophilaenus*, potentially causing serious disruptions to forest ecosystem structure and function [65].

*X. fastidiosa* is a paradigm of vascular phytopathogens, with biofilm formation directly driving disease manifestation. The bacterium colonises xylem vessels, producing an EPS matrix that contributes to vessel blockage and reduced sap flow; this effect is a primary determinant of wilting and shoot dieback symptoms in many hosts, including grapevine, olive, and citrus [66]. Key elements for pathogenicity include cell-surface adhesins, filamentous EPS structures, and DSF (diffusible signal factor) signalling systems, which regulate the balance between adhesion (biofilm) and dispersion (planktonic phase) and influence vector-mediated transmission. Interventions that modulate DSF signalling or degrade EPS have been shown to reduce biofilm formation and lower virulence in experimental models [12,67].

Recent studies have combined biofilm imaging of *Xylella* in vitro and in planta with modelling of flow mechanics in infected vessels, providing insights into how biomass accumulation and EPS rheological properties lead to impaired plant hydraulic function. These findings highlight that controlling biofilm in *X. fastidiosa* may require a combination of approaches, from modulation of communication signals to enzymatic matrix disruption [68].

Establishment of xylem-colonising, biofilm-forming pathogens such as *X. fastidiosa* in natural forest ecosystems would likely produce a range of ecological and socio-economic effects that extend far beyond direct mortality of individual host trees. First, by selectively reducing vigour or killing susceptible canopy trees, chronic vascular colonisation can alter stand structure and reduce habitat heterogeneity, with downstream losses in species that depend on mature trees (e.g., cavity-nesting birds, saproxylic invertebrates and specialised epiphytes), thus decreasing local biodiversity and genetic diversity of host populations [69]. Second, widespread xylem blockage and elevated tree mortality change the input and quality of woody litter, with consequences for decomposition pathways and nutrient cycling. Increased deadwood inputs and altered litter chemistry can shift soil microbial communities and decomposition rates, modifying carbon and nutrient fluxes at stand and landscape scales and potentially affecting long-term soil fertility and carbon sequestration [70,71]. Third, the formation of persistent infection foci and recurring dieback can redirect successional trajectories. Canopy gaps created by mass decline may favour opportunistic, early-successional species or invasive plants, altering species composition and potentially reducing native forest resilience. Such shifts in succession can be amplified where combined stresses (drought, pests) interact with pathogen pressure [72]. Fourth, ecosystem services provided by forest stands—including water regulation, erosion control, carbon storage, cultural services and non-timber forest products—may be diminished locally or regionally if large areas are impacted. Economic impacts will not be limited to timber losses: they include costs of monitoring, sanitary measures, removal of infected trees, loss of recreation/tourism value, and broader effects on landscape-level provisioning services previously documented for agricultural tree systems and mixed landscapes affected by *Xylella* [71]. Finally, from an epidemiological and management perspective, biofilms in trees complicate detection and control: internal, matrix-protected bacterial populations are less accessible to treatments and may require long-term monitoring and extensive sanitary interventions, substantially raising management costs. The combination of ecological cascades (biodiversity loss, altered nutrient cycling and succession) and high socio-economic costs argues for proactive surveillance, landscape-scale risk assessment and integrative management approaches that account for forest-specific anatomy, microbiomes and ecosystem functions [70,71].

### 3.4. Brenneria goodwinii and Gibbsiella quercinecans—Acute Oak Decline (AOD) and the Biofilm Synergy Hypothesis

The bacterial complex associated with Acute Oak Decline (AOD) was first identified in the United Kingdom in the mid-2000s and is now recognised as a serious threat to the health of oaks in Europe. The disease primarily affects *Quercus robur* and *Q. petraea*, and less frequently *Q. cerris*, and is characterised by bark cracks, oozing of dark fluid, and necrosis of subcortical tissues [73]. The AOD microbial complex mainly includes *B. goodwinii*, *G. quercinecans*, and *Rahnella victoriana*, often co-occurring with xylophagous insect larvae such as *Agrilus biguttatus*, which may facilitate bacterial entry into the wood tissue [59,74]. These bacteria have also been detected in oak populations across many parts of Europe [75,76,77,78]. Although the mechanisms of pathogenesis are not fully understood, numerous isolation studies suggest that biofilm formation may play a significant role in stabilising infections and promoting tissue necrosis in AOD.

The bacterial consortium associated with AOD, particularly *B. goodwinii*, *G. quercinecans*, and other co-isolated species, represents a complex, multi-species microbiota interacting in tree pathogenesis. Increasing evidence from isolation and metagenomic studies identifies these species at AOD sites, and co-cultivation experiments suggest interactions that may enhance mutual adaptability and the ability to colonise wounds and necrotic tissue [79]. A hypothesis emerging in the literature proposes that biofilm formation as a trait of the bacterial consortium may support the stabilisation of infection foci in areas of bark and vascular tissue necrosis by joint EPS production, protection against host defences, and facilitation of cooperative tissue degradation [75,80].

Unfortunately, direct microscopic studies of biofilms in oak tissues remain scarce; most evidence relies on species isolation, in vitro virulence assays, and pathological observations. This methodological gap—the lack of in situ visualisation and quantitative EPS analyses in tree samples—represents an important avenue for future research. Adapting techniques such as CLSM, FISH, X-ray microtomography, and EPS chemical analyses to woody tissues could verify the hypothesis of biofilm as a synergistic component in AOD [80].

To transform the biofilm synergy hypothesis into a validated theory, direct experimental evidence demonstrating cooperative biofilm formation in situ within oak tissues is required. The most convincing data would include (i) microscopic visualisation of mixed-species biofilms containing *B. goodwinii*, *G. quercinecans*, and associated taxa within necrotic xylem and bark; (ii) co-cultivation or microcosm experiments showing enhanced biofilm biomass, EPS production, or virulence when species are combined compared to monocultures; and (iii) metatranscriptomic or metabolomic evidence of interspecies signalling or metabolic complementarity within the biofilm matrix [73,81]. Such evidence would directly link microbial cooperation to tissue degradation and disease progression.

At present, the main limitation is predominantly technical rather than biological. Most AOD-associated bacteria are cultivable under standard conditions, but in situ biofilm visualisation in woody tissues remains challenging due to tissue opacity, lignification, and the difficulty of applying fluorescent markers or probes deep within the wood matrix [82]. Adapting methods such as confocal laser scanning microscopy (CLSM) combined with fluorescence in situ hybridisation (FISH) or micro-computed tomography (micro-CT) to hardwood samples is essential for spatial verification of mixed-species biofilms. However, additional biological complexity exists: multi-species interactions may be context-dependent, requiring environmental cues or insect-vectored inoculation to trigger cooperative EPS production [83]. Thus, the experimental barrier is dual—primarily technical in visualisation, but secondarily biological in replicating the ecological context necessary for biofilm synergy to emerge.

### 3.5. L. populi—Biofilm-Driven Pathogenesis in Poplar Trees

*L. populi* has emerged as a major bacterial pathogen of Populus species across Europe and Asia, causing extensive canker and dieback symptoms in both natural and plantation stands. Over the last decade, field surveys in China, Hungary, Portugal, Spain and Central Europe have reported a steady increase in infection frequency, correlating with climatic stress and insect activity [84,85,86].

A distinctive feature of *L. populi* infection is the production of a whitish, creamy biofilm-like exudate on bark fissures and wound margins. This exudate consists of abundant extracellular polymeric substances (EPS) rich in polysaccharides and proteins, forming a viscous matrix that protects bacterial cells from desiccation and UV radiation. The biofilm structure also enhances bacterial aggregation and persistence on bark surfaces, facilitating survival under fluctuating humidity conditions typical of riparian and plantation habitats [87].

Recent microscopic and biochemical analyses [60,87,88] confirm that the EPS of *L. populi* acts as a key virulence factor. It supports adhesion to xylem parenchyma, impedes host phenolic diffusion, and mediates cell-to-cell communication through quorum sensing signals analogous to AHL-type autoinducers. Notably, the biofilm exudate appears to attract insect vectors, promoting bacterial dissemination between trees—a rare ecological linkage between microbial virulence and insect-mediated spread in forest systems.

Physiologically, *L. populi* shares certain features with *E. amylovora* and *B. goodwinii*, including the production of exopolysaccharides and secretion of cell wall-degrading enzymes. However, unlike vascular pathogens such as *X. fastidiosa*, *L. populi* primarily colonises cortical and subcortical tissues, where biofilm protects against oxidative plant defences. This mixed strategy—epiphytic persistence combined with localised necrosis—underscores the ecological flexibility of *L. populi* and highlights the need to integrate this species into forest biofilm pathogenesis frameworks [15,87].

As summarized in Table 2, the strength of evidence for biofilm involvement varies greatly among tree pathogens, ranging from well-documented cases such as *X. fastidiosa* to emerging examples like L. populi. This overview emphasizes the need for further in situ research to clarify the ecological roles of biofilms in forest disease dynamics.

## 4. Biofilm as a Target in Tree Disease Management Strategies

Advancing understanding of the role of biofilms in the pathogenesis of tree-associated bacteria has opened a new direction in plant health management: biofilm-targeted control. This approach focuses on disrupting biofilm formation or maintenance. Unlike traditional methods that target planktonic bacterial cells, these strategies aim to disorganise biofilm structure, interrupt cellular communication (quorum sensing, QS), or degrade the EPS matrix, thereby increasing pathogen sensitivity to environmental stresses and plant protection measures [92].

### 4.1. Chemical Biofilm Inhibitors

Chemical biofilm inhibitors primarily target quorum sensing (QS) signalling and degrade the EPS matrix. Many natural and synthetic compounds, such as halogenated furanones, phenylacetic acids, cinnamaldehyde, and gallate derivatives, have been shown to disrupt QS communication in phytopathogenic bacteria, including *P. syringae* and *X. fastidiosa* [93,94]. These compounds act by blocking LuxR- or RpfC-type receptors, thereby inhibiting the expression of genes responsible for EPS synthesis, biosurfactants, and virulence enzymes [95].

Parallel enzymatic approaches target structural components of the biofilm. DNases, alginases, and bacterial amylases destabilise biofilms by hydrolysing extracellular DNA or EPS polysaccharides [22]. In models of *E. amylovora* and *P. syringae*, application of these enzymes reduced adhesion and increased bacterial susceptibility to biocides. Moreover, combining enzymes with low doses of copper or silver can synergistically enhance anti-biofilm efficacy [96].

### 4.2. Biological Approaches

Biological strategies to limit biofilm formation by tree-pathogenic bacteria rely on antagonistic microorganisms, endophytes, and natural secondary metabolites, which can interfere with adhesion, intercellular communication, or the structural stability of biofilms. A well-studied model involves Bacillus strains, which produce biosurfactants, particularly surfactin, that effectively reduce biofilm formation in *Pseudomonas* species. As demonstrated by Bais et al. [97], *Bacillus subtilis* 6051 colonising the roots of *Arabidopsis thaliana* forms a protective biofilm that acts as both a physical and chemical barrier against *P. syringae* pv. *tomato* DC3000. Mutants unable to synthesise surfactin lost this protective ability, highlighting the key role of biosurfactants in biocontrol.

Other *Bacillus* species, such as *B. halotolerans*, produce surfactin and fengycin with strong antimicrobial activity and the ability to destabilise pathogen biofilms [98]. Endophytic strains, such as *Bacillus pumilus* 2A isolated from plant tissues, produce amphiphilic biosurfactants that inhibit pathogenic colonisation and promote plant growth, demonstrating both bioprotective and biostimulatory potential [99].

Enzymes and bacterial metabolites capable of degrading biofilm matrix components represent another important approach. For example, PslG_PF glycoside hydrolase from *P. fluorescens* effectively breaks down biofilm exopolysaccharides and inhibits biofilm formation in related species, including *P. syringae* [100]. Such enzymes could serve as eco-friendly biocontrol additives to prevent tree infections.

Another mechanism is quorum sensing degradation (quorum quenching). Newman et al. [101] showed that bacteria from the *Bacillus*, *Paenibacillus*, *Microbacterium*, and *Pseudomonas* genera can degrade DSF (diffusible signal factor) molecules, significantly reducing virulence and biofilm formation in pathogens such as *X. fastidiosa* and *Xanthomonas campestris*. This approach is particularly valuable for xylem-inhabiting pathogens, where conventional chemicals have limited access.

Finally, microbiome interference—introducing or supporting endophytic and epiphytic microbes that compete with pathogens for space and resources—is gaining attention. Certain *P. fluorescens* strains can form non-pathogenic biofilms on leaf surfaces, limiting pathogen colonisation while stimulating plant immunity [102]. In forest trees, selective inoculation of such strains could provide an ecologically safe method to stabilise bark and leaf microbiomes and suppress biofilm-forming pathogens.

The use of antagonistic microorganisms such as *P. fluorescens* and *Bacillus* spp. in forest ecosystems poses ecological risks due to the complexity of native microbial networks [103]. Introducing non-native strains can disrupt mutualistic fungi or nutrient-cycling communities and may act unpredictably outside their original host context. Effective and safe application therefore requires host-adapted isolates, ecological compatibility testing, and long-term monitoring to avoid unintended ecosystem effects.

### 4.3. Environmental Management

From a forest ecology perspective, indirect measures aimed at maintaining microbial balance and limiting factors that favour the planktonic phase of bacteria can play a key role in biofilm control. Maintaining a healthy soil and bark microbiome by reducing mechanical disturbances, avoiding excessive nitrogen fertilisation, and promoting saprotrophic microorganisms can help lower pathogen pressure [104].

Microclimate management—particularly reducing excessive moisture and water stagnation near the bark—can reduce conditions that trigger adhesion gene activation and biofilm formation [105]. In nurseries and plantations, drip irrigation is often preferred over overhead spraying, as it limits the spread of planktonic forms of *Pseudomonas* and *Erwinia* [106].

The implementation of biofilm-targeted control in large-scale forest ecosystems faces significant practical constraints. Unlike managed orchards or nurseries, forests are structurally complex and only partly accessible, making the targeted delivery of quorum-quenching agents or biosurfactants technically difficult. Environmental heterogeneity, canopy interception, and the absence of irrigation or containment systems further reduce treatment efficiency. Promising future directions include biologically based delivery systems—such as non-pathogenic endophytes capable of producing quorum-quenching enzymes—and localized slow-release formulations applied to high-risk sites. Nevertheless, ecological safety, persistence, and scalability remain major challenges that must be addressed before field-level application becomes feasible [12,22,97,101].

### 4.4. Management Tools for Established Xylem-Pathogens and Endophyte Inoculation

When xylem-colonizing pathogens become established within the vascular tissues of forest trees, traditional interventions (such as chemical trunk injections or foliar sprays) often lose much of their effectiveness because the pathogen resides within the inaccessible xylem network. At this stage forest practitioners commonly face two main options: removal of infected trees or acceptance of disease progression. However, a promising complementary route is the enhancement of beneficial endophytic microorganisms within the woody tissues, which may help to suppress pathogen activity, stimulate host resistance and maintain tree vitality [22,28]. Research on bacterial endophytes in forest trees [107,108] shows that genera such as *Pseudomonas*, *Bacillus* or *Paenibacillus* colonize internal tissues of long-lived tree species and confer benefits including growth promotion and pathogen antagonism.

In practice, inoculation of endophytes may be achieved in plantation or nursery-raised seedlings prior to out-planting, thereby establishing a beneficial microbiome before pathogen invasion. For mature stands, delivery remains challenging—possible approaches include trunk injection of endophytic consortia, root-zone drenches combined with mycorrhizal-based carriers, or stem coatings that allow endophyte uptake. Nevertheless, limitations persist: host-specificity of endophytes, competition with native microbiota, environmental variability and monitoring cost are non-trivial barriers [109,110].

Thus, integrating endophyte-based strategies into forest disease management requires a shift toward preventive establishment in the nursery phase, followed by ecosystem-scale delivery systems for mature stands. Such integration also demands rigorous monitoring of colonisation success, tree response and long-term persistence of the introduced microbiota [97]. The key application pathway is: (i) Nursery inoculation of seedlings with selected endophytes → establishment of microbiome prior to planting, (ii) monitoring of seedlings for endophyte colonisation and health status, (iii) in standing forests: targeted delivery (injection, drench, coating) of endophytes into high-value trees showing early signs of xylem-pathogen infection, and (iv) long-term monitoring of tree performance, microbiome stability and disease suppression.

This two-tier approach (nursery + mature stand intervention) may help mitigate the challenge of pathogen control once the xylem colonisation is advanced, and offers an ecosystem-oriented alternative to the “remove or accept” paradigm.

## 5. Perspectives and Challenges

Despite the growing body of research on plant-pathogenic bacterial biofilms, in situ studies in forest stands remain scarce. Most data are derived from laboratory or nursery conditions, whereas the natural forest environment—with its fluctuating humidity, temperature gradients, and heterogeneous microbiomes—is not sufficiently represented [111,112]. This limitation restricts our ability to predict the actual dynamics of biofilms, their survival strategies, and their role in tree diseases.

Technically, visualising biofilms inside wood, especially within xylem vessels, is challenging due to lignification, tissue heterogeneity, and limited access for light or fluorescent dyes. In many cases, methods such as confocal laser scanning microscopy (CLSM) or FISH must be adapted or replaced by imaging approaches such as microtomography. For example, Castro et al. [8] demonstrated that β-1,4-endoglucanase affects EPS polymer length and biofilm dynamics, but these experiments were conducted primarily in vitro, highlighting the gap in studies under natural tree conditions.

There is significant potential to translate knowledge from agricultural systems to forests, while accounting for tree-specific traits. One example is the study using xylem sap from Vitis riparia as a natural substrate to grow *X. fastidiosa*, which helped to better understand biofilm architecture under conditions approximating natural plant vessels [113]. Such approaches enable the adaptation of biological, genetic, and ecological models to forest trees, which vary in wood structure, tissue density, natural barriers (e.g., suber, cork, cambial boundaries), and other abiotic and biotic factors.

Another critical aspect is the development of biofilm imaging methods in trees. Micro-computed tomography (micro-CT) is increasingly used for visualising tissue structures and can be adapted to observe xylem vessels and biofilm niches [112]. For instance, a study employing 3D micro-CT to visualise part of the digestive tract of *X. fastidiosa* insect vectors demonstrates the potential of microtomography in biofilm biology [114]. Although not performed in trees, this illustrates technical possibilities that could be transferred to forest systems. Emerging analytical tools such as correlative light and electron microscopy (CLEM), Raman–FISH spectroscopy, and biosensor-based detection of quorum-sensing signals provide new opportunities to study biofilms in situ in woody tissues. These methods can reveal the spatial organisation and metabolic activity of bacterial consortia beyond the limits of micro-CT and CLSM, offering a powerful complement for future forest pathology research [92].

**Figure 1:**
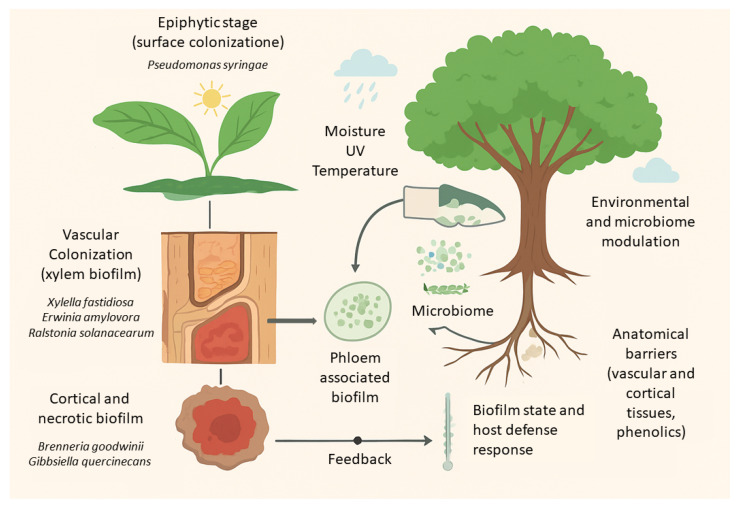
Integrative model of biofilm-mediated infection in forest tree pathogens. Three principal biofilm strategies—epiphytic, vascular, and cortical/necrotic—are shown along a gradient of tissue colonization. The model incorporates environmental modulators (temperature, humidity, oxidative stress), the influence of the tree microbiome, and anatomical barriers such as xylem structure and bark thickness. Arrows indicate feedback interactions between pathogen biofilm dynamics and host defence responses, illustrating potential points for biofilm-targeted intervention.

A conceptual model integrating biofilm into the life cycle of tree pathogens could be developed (Figure 1). Achieving this, however, requires overcoming several challenges: development of non-invasive imaging methods, such as micro-CT adapted to wood fragments; standardisation of sample collection and preparation for metagenomic and microscopic analyses; identification of biofilm markers specific to tree species; and experimental validation of the model in natural forest populations.

From an evolutionary perspective, describing biofilms as complex adaptive systems highlights their collective ability to respond dynamically to selective pressures exerted by long-lived hosts and fluctuating environments. Within biofilms, bacterial populations act as quasi-multicellular entities capable of sharing resources, exchanging genetic material, and regulating gene expression cooperatively through quorum sensing [92,115]. This collective organisation increases phenotypic plasticity and accelerates adaptation to host defences, effectively buffering individual cells against selective sweeps. In the context of long-lived forest trees, such cooperative resilience allows pathogens to persist across multiple host generations, maintaining evolutionary continuity and promoting the gradual refinement of virulence strategies. Conversely, trees respond by reinforcing structural and chemical defences—such as phenolic deposition, compartmentalisation, and microbiome-mediated exclusion—resulting in a prolonged coevolutionary arms race. Thus, biofilm formation not only serves as an ecological survival strategy but also as an evolutionary mechanism that stabilises pathogen–host interactions over extended temporal scales.

## 6. Summary

Biofilms are a key, though long underappreciated, component of the survival and virulence strategies of bacterial pathogens in forest trees. Numerous studies and literature reviews indicate that the ability to form biofilms is widespread among phytopathogenic bacteria, regardless of their ecological niche or infection strategy. Both epiphytic surface-associated species, such as *P. syringae*, and vascular internal pathogens, including *Xylella fastidiosa* and *E. amylovora*, use biofilm forms as a central mechanism enabling stable host colonisation, increased resistance to environmental stresses, and more effective evasion of plant defence mechanisms. In all these cases, the biofilm serves both protective and pathogenic functions, shaping the course of infection and influencing disease dynamics in forest ecosystems.

In forest environments, biofilm formation is particularly significant due to the complexity and variability of ecological conditions. Tree species diversity, microclimatic heterogeneity, and the presence of rich endophytic and epiphytic microbiomes create niches in which biofilms become highly effective adaptive strategies. Biofilms allow prolonged bacterial survival on leaf and bark surfaces as well as within vascular tissues, forming the basis for chronic infections that are difficult to control. The recently described *L. populi* exemplifies a bark-colonising, biofilm-producing tree pathogen whose EPS exudates mediate both protection and vector attraction, highlighting novel ecological dimensions of biofilm function in forest systems. This phenomenon is especially relevant in diseases such as Acute Oak Decline (AOD), where biofilms may act as a stabilising factor in necrotic tissue microenvironments and facilitate synergy among co-occurring bacterial species.

The formation and maintenance of biofilms is a complex, multilayered process. Quorum sensing systems, cyclic-di-GMP signalling pathways, and transcriptional regulatory networks govern transitions between planktonic and sessile states, determining bacterial population behaviours in response to environmental cues and host interactions. Understanding these mechanisms is fundamental for future tree protection strategies, as the regulation of intercellular communication and EPS matrix synthesis largely dictates biofilm resistance to biocides and environmental stressors.

Despite significant advances in the study of plant bacterial biofilms, their role in forest ecosystems remains poorly understood. In situ studies allowing direct visualisation and analysis of biofilms in tree tissues—especially xylem and phloem—are lacking. The complex wood structure, lignification of tissues, and limited accessibility of imaging techniques present major methodological challenges. However, the development of advanced microscopy, microtomography, spectroscopy, and metagenomic analyses provides an opportunity to overcome these limitations and deepen knowledge of biofilm biology under natural forest conditions.

Understanding biofilm function in tree pathogenesis also opens new perspectives for phytosanitary practice. Increasing attention is being given to “biofilm-targeted control” strategies, which do not focus on eliminating planktonic bacteria, but rather on disrupting biofilm structures, blocking quorum sensing signals, or enzymatically degrading the EPS matrix. These approaches, combined with the use of antagonistic microorganisms and naturally derived biosurfactants, may offer an ecologically safe alternative to conventional chemical treatments, particularly in forest settings where interventions should be minimally invasive.

In conclusion, biofilms are not merely a physiological form of bacterial organisation, but a complex adaptive system crucial for the pathogenesis, survival, and evolution of bacteria in forest trees. Their presence affects the course of infection, disease dynamics, and the microbiological stability of forests. Integrating microbiological, ecological, and technological knowledge in biofilm research provides new opportunities to develop effective, sustainable strategies for maintaining tree health and preserving the biological balance of forest ecosystems.

## Figures and Tables

**Table 2:** Strength of evidence for biofilm involvement in major tree pathogens. Strength of evidence: +++ strong, multi-level evidence (laboratory, host, and field); ++ moderate, mainly experimental evidence; + preliminary, limited or indirect observations.

Pathogen	Types of Evidence (In Vitro/In Planta/In Situ)	Strength of Evidence (+++/++/+)	Key Knowledge Gaps	References
*X. fastidiosa*	Robust in vitro and in planta evidence; multiple in situ visualizations using CLSM and SEM; well-characterized genetic regulation (rpf, DSF)	+++	Long-term field quantification of EPS and vessel occlusion in forest hosts	[23,24,34]
*E. amylovora*	Extensive in vitro and in planta biofilm assays; QS and c-di-GMP systems described; few in situ forest data (mostly orchards)	++	Field verification of biofilm formation in wild forest hosts	[15,54]
*P. syringae*	Strong in vitro and in planta assays; phyllosphere imaging; indirect in situ evidence of overwintering biofilms	++	Quantitative in situ microscopy on forest trees; biofilm dynamics under natural conditions	[89]
AOD complex	Several co-culture and metagenomic studies; indirect in situ support from microscopy and pathology correlations	+/++	Direct visualization and EPS composition analyses in situ; confirmation of cooperative biofilm physiology	[59,74]
*L. populi*	Field observations and isolation data; limited in vitro and no in planta quantification yet; reports of EPS composition and QS signals emerging	+ (emerging)	Controlled in planta infection and microscopy; biochemical characterization of EPS; demonstration of insect-mediated spread	[90]
Ralstonia solanacearum (tree-associated lineages)	In vitro and in planta vascular colonization studies; in situ micro-CT visualization in Eucalyptus	++	Long-term ecological dynamics in forest hosts; QS signaling variability	[49]
*Xanthomonas spp.* (e.g., *X. arboricola, X. campestris*)	Strong in vitro assays and crop data; limited in situ forest imaging	+/++	Validation of host-specific biofilm mechanisms in natural forests	[91]

## Data Availability

No new data were created or analyzed in this study. Data sharing is not applicable to this article.
